# Comparison of rocuronium priming vs. standard rapid sequence intubation technique in emergency department patients requiring intubation

**DOI:** 10.1590/1806-9282.20231029

**Published:** 2024-04-22

**Authors:** Nurullah İshak Işık, Ayhan Özhasenekler, Çağdaş Yıldırım, Alp Şener, Fatih Ahmet Kahraman, Gül Pamukçu Günaydın

**Affiliations:** 1 Ankara Etlik City Hospital, Department of Emergency Medicine – Ankara, Turkey.; 2 Ankara Bilkent City Hospital, Department of Emergency Medicine – Ankara, Turkey.

**Keywords:** Airway management, Rapid sequence induction and intubation, Emergency medicine, Rocuronium

## Abstract

**OBJECTIVE:**

In our study, we aimed to compare the effect of standard rapid sequence intubation protocol and the application of rocuronium priming technique on the procedure time and hemodynamic profile.

**METHODS:**

Patients who applied to the emergency department and needed rapid sequence intubation were included in our study, which we conducted with a randomized controlled design. Randomization in the study was made according to the order of arrival of the cases. Rapid sequence intubation was performed in the standard group. In the priming group, 10% of the rocuronium dose was administered approximately 3 min before the induction agent. Intubation time, amount of drug used, vital signs, and end-tidal CO_2_ level before and after intubation used to confirm intubation were recorded.

**RESULTS:**

A total of 52 patients were included in the study, of which 26 patients were included in the standard group and 26 patients in the priming group. While intubation time was 121.2±21.9 s in the standard group, it was calculated as 68.4±11.6 s in the priming group (p<0.001). While the mean arterial pressure was 58.3±26.6 mmHg in the standard group after intubation, it was 80.6±21.1 mmHg in the priming group (p=0.002).

**CONCLUSION:**

It was observed that priming with rocuronium shortened the intubation time and preserved the hemodynamic profile better.

**Clinical Trial Registration Number:**

NCT05343702.

## INTRODUCTION

Rapid sequence intubation (RSI) is a commonly used technique for elective intubation, aiming to provide optimal physiological conditions while carrying some inherent risks such as hypotension, hypoxia, and aspiration of gastric content^1,2^. The primary goal of RSI is to achieve the intubation procedure as quickly as possible, minimizing complications in the subsequent process^1^. Emergency department patients often undergo this procedure without prior evaluation, which may increase the likelihood of complications. Although there is procedural consensus on measures to prevent gastric aspiration and hypoxia, clear recommendations for managing hypotension are currently lacking^1–4^.

The advent of rocuronium, a non-depolarizing neuromuscular agent (NMBA), has increased its popularity, making it one of the most widely accepted agents for RSI^5^. The priming technique, first proposed by Foldes, has been shown in numerous studies to expedite the onset of NMBA action during intubation^6,7^. Theoretically, a small preceding dose of NMBA administered before induction leads to faster receptor stimulation and quicker onset of action.

Although the priming technique is generally considered hemodynamically neutral, the existing literature mainly comprises pre-evaluated patients in operating rooms and intensive care units. Unfortunately, there is a lack of studies focusing on emergency department patients^8^.

In our study, we aimed to investigate the impact of rocuronium priming technique compared with the standard RSI protocol on intubation times and hemodynamic responses in patients requiring intubation upon presentation to the emergency department.

## METHODS

Our study was conducted between July 15, 2021, and December 01, 2021, at the Ankara City Hospital Emergency Department, which is a tertiary care center with approximately 450,000 annual patient admissions. The study design was prospective and randomized, and it received ethical approval from the Ankara City Hospital Ethics Committee 2 (Ethical Approval No. E2-21-631). Written consent was obtained from all patients, and the study was registered on clinicaltrial.gov (No. NCT05343702).

The inclusion criteria for our study were as follows:

- Age 18 years and above;
- Requirement for advanced airway management;
- Written consent from the patient or consent from the patient's relatives if the patient was unable to provide consent.

The exclusion criteria were as follows:

- Presence of crush (rescue) airway indication;
- Meeting any of the LEON criteria, indicating a difficult airway;
- Ineligibility for ketamine administration due to clinic-related reasons or allergy.

Data were collected using a data collection form, which included information on patients’ age, gender, presence of diabetes mellitus, indication for intubation, vital signs (i.e., blood pressure, pulse rate, and oxygen saturation) before and 10 min after intubation, intubation duration, drug dosages used, end-tidal CO_2_ level for intubation confirmation, number of successful attempts for intubation, and the need for alternative airway management. We only recorded diabetes mellitus as a chronic disease because diabetes patients have a higher likelihood of prolonged neuromuscular blockade^9^. The selection of the initial and 10th-min vital signs was based on the assumption that the physiological response to intubation (resulting from intense sympathetic and parasympathetic stimulation in the upper airway) would almost return to normal by the 5th min, while the effect of rocuronium would still be present, leading to more accurate results for the primary outcomes of our study^10^. For randomization, patients included in the study were assigned to the standard technique group if their admission number was odd and to the priming technique group if their admission number was even.

Our study's power analysis followed Şen et al.'s approach, which utilized onset time of action data for "Depolarizing and non-depolarizing neuromuscular agents." To achieve 99% power and a 5% type-1 error rate, each group required a minimum of 21 patients^11^.

A common induction agent and a neuromuscular blocking agent (NMBA) were used for both groups. Ketamine was used as the induction agent with a dosage of 1 mg/kg, while rocuronium was used as the NMBA with a total dosage of 1 mg/kg. Chin relaxation was considered the indication of NMBA effectiveness in both groups. The start of the stopwatch was considered the moment when ketamine administration began for both groups. The end of the stopwatch was considered when the tube passed through the vocal cords, and the operator declared "I have passed." The onset of action of ketamine was considered 30 s for both groups. Capnometry was used to measure end-tidal carbon dioxide levels for verification after the intubation procedure. Vital signs were measured again at the 10th minute after the procedure. The standard technique followed the RSI protocol, while the priming technique involved administering 10% of the total planned rocuronium dose before induction and the remaining dose after the onset of action of the induction agent.

Our primary outcomes were intubation duration and hemodynamic response. Statistical analyses utilized SPSS for Windows 22.0. Normality was assessed with the Shapiro-Wilk test. Normally distributed data were presented as mean, standard deviation, and 95% confidence interval, and non-normally distributed data were shown as median, interquartile range, minimum, and maximum. Categorical variables were compared using Pearson chi-square and Fisher's exact tests. The independent-samples t-test assessed normally distributed continuous data for independent group comparisons and the paired-samples t-test for dependent group comparisons. The Mann-Whitney U test analyzed non-normally distributed continuous data for independent groups and the Wilcoxon signed-rank test for dependent groups. Significance was set at p<0.05.

## RESULTS

**Figure 1:**
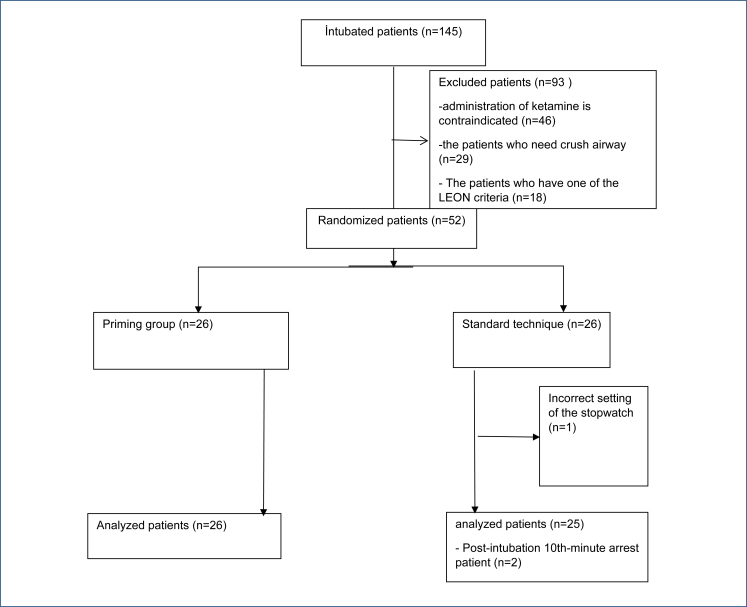
Flow diagram.

After applying the exclusion criteria, a total of 52 patients were initially included in the study. However, data from one patient were excluded due to missing information. Consequently, the study was completed with 26 patients in the priming group and 25 patients in the standard group, and their data were analyzed (Figure 1).

There were no statistically significant differences between the groups in terms of mean age, gender distribution, and presence of diabetes among the included patients. Analysis of the pre-intubation hemodynamic profiles of the patients also revealed similar characteristics and no statistically significant differences between the two groups (Table 1).

**Table 1:** Demographic characteristics and vital signs.

Variables		Standard group (n=25)	Priming group (n=26)	p-value
Age median (IQR)		80.0 (70.0–84.0)	79.0 (69.0–88.0)	0.806
**Sex**				
	Male (%)	13 (52.0)	15 (57.7)	0.683
	Female (%)	12 (48.0)	11 (42.3)	0.683
Diabetes mellitus (%)		9 (36.0%)	8 (30.8%)	0.692
SBP median (IQR) mmHg		110 (96–131)	110 (101–135)	0.434*
DBP median (IQR) mmHg		62 (53–77)	64.5 (56–75)	0.699†
MAP median (IQR) mmHg		84.3 (66.0–92.0)	79.7 (71.3–96.7)	0.378†
Heart rate (IQR) pulse/min		115 (98–139)	106 (94–118)	0.511*
SO_2_ median (IQR) %		86 (78–92)	81,5 (71–89)	0.163*

SBP: systolic blood pressure; DBP: diastolic blood pressure; MAP: mean arterial pressure; IQR: interquartile range.

* Independent-samples t-test.

† Mann-Whitney U test.

When looking at the primary outcome, which is the intubation duration, it was found that the priming group had a significantly shorter intubation duration (Table 2). Another primary outcome, hemodynamic stability, was examined, and it was observed that the priming group exhibited a more stable hemodynamic profile at the 10th-min post-intubation. When analyzing the data at the 10th-min post-intubation, the priming group had higher values for systolic blood pressure, diastolic blood pressure, and mean arterial pressure compared with the standard group, and there was a statistically significant difference between the groups. However, there was no statistically significant difference between the groups regarding pulse rate and oxygen saturation (Table 2).

**Table 2:** Post-intubation hemodynamic parameters and intubation times.

	Standard median (IQR)	Priming median (IQR)	p-value
Intubation time	117.0 (107.0–132.0)	67.5 (59.0–76.0)	<0.001†
Post SBP (mmHg)	80 (60–105)	104.5 (85–137)	0.007*
Post DBP (mmHg)	50 (32–60)	61 (53–75)	0.001*
Post MAP (mmHg)	59.7 (41.7–73.7)	72.7 (64.7–89.0)	0.002†
Heart rate (pulse/min)	124 (95–135)	106.5 (97–135)	0.659*
Post SO_2_ (%)	95 (87–97)	93.5 (90–97)	0.762†

* Independent-samples t-test.

† Mann-Whitney U test.

When examining the success of intubation based on the groups, there was no statistically significant difference between the groups. The median number of attempts in the priming group was 1.0 (mean value), while in the standard group, it was also 1.0 (p=0.362).

Regarding the verification of intubation placement, we analyzed the post-intubation end-tidal CO_2_ values. The median value in the priming group was 26.0 mmHg (interquartile range: 20.0–29.0), while in the standard group, it was 29.0 mmHg (interquartile range: 24.0–59.0). There was a statistically significant difference between the groups (p=0.029).

## DISCUSSION

When examining the results of our study, it was found that the priming technique resulted in a significantly shorter intubation duration and a more stable hemodynamic profile compared with the standard RSI.

As known, certain complications during intubation can lead to various undesired outcomes for patients in the short and long term. For instance, hypotension is a feared occurrence during intubation in both emergency services and urgent surgical cases, particularly for patients with no fasting period. Hypotension develops in almost one-fourth of patients, and this subgroup is associated with a higher mortality rate^12^. Our study designated these two issues as the primary outcomes and demonstrated that the priming technique was more beneficial.

When reviewing the literature on priming dose, it is evident that most studies have been conducted by anesthesiologists. Our study stands out as the first one conducted in the emergency department setting. Notably, the distribution of characteristics, including age, gender, and additional features, was similar between the groups. Our study includes older patients, with an average age of 79 years, which is not commonly seen in the literature^13,14^. Some studies suggest that the priming technique should not be applied to elderly patients or those with poor lung reserves^8,15^. However, we could not find any specific recommendations in the literature pertaining to our patient population.

It is well-known that the priming technique reduces intubation duration, and several studies have provided evidence supporting this finding^7^. The results of our study align with the literature in this regard. Moreover, being the first study conducted specifically in the emergency department setting and involving a higher proportion of elderly patients adds to its distinctiveness. Regarding hemodynamic stability, rocuronium is among the best nondepolarizing neuromuscular agents, and it is generally accepted that priming application does not impact the hemodynamic response^16^. There is a wealth of data on intubation-related hypotension, which can be influenced by various factors such as the physiology of intubation, drugs used, and the patient's clinical condition (e.g., hypovolemia and shock). Additionally, there are articles in the literature indirectly investigating the relationship between rocuronium use and hypotension^17^. When examining the literature, we notice a common feature of planned RSI performed in low-risk, young patients. In contrast, our study involves patients with relatively unstable conditions in the emergency department, including older patients. The age-related differences in rocuronium pharmacokinetics and the fragility of these patients might have contributed to the emergence of a significant difference. This suggests that priming dosage might be more beneficial for this critical patient group. Furthermore, the higher end-tidal CO_2_ values in the priming group could confirm better perfusion in these patients.

In conclusion, our study's findings indicate that the priming technique results in a significantly shorter intubation duration and a more stable hemodynamic profile compared with the standard technique. These results hold potential importance for critical patients in the emergency department. However, further studies with larger patient populations and diverse settings are needed to validate these findings.

## LIMITATIONS

One of the main limitations of our study is that it was single-blinded. We attribute this to the fact that the study was conducted in a large hospital with intubations performed by various personnel in multiple areas. To overcome this limitation, we ensured that the data collection process was carried out by an independent individual.

## CONCLUSION

Our study demonstrated that the priming technique significantly reduced intubation duration and resulted in a more stable and consistent hemodynamic profile in terms of systolic blood pressure, diastolic blood pressure, and mean arterial blood pressure.
